# Unexpected Culprit: Unveiling a Unique Case of Appendicitis Triggered by a Foreign Object

**DOI:** 10.7759/cureus.60910

**Published:** 2024-05-23

**Authors:** Thaanesh Sankar, Gaurav Dhoka, Yashaswinii P, Guru Prasad, Karthik Krishna Ramakrishnan

**Affiliations:** 1 Department of Surgery, Sree Balaji Medical College and Hospital, Chennai, IND; 2 Department of Radio-Diagnosis, Saveetha Medical College and Hospital, Saveetha Institute of Medical and Technical Sciences, Saveetha University, Chennai, IND

**Keywords:** surgical abdominal emergency, fish bone perforation, surgical appendectomy, foreign body, traumatic appendicitis

## Abstract

Appendicitis is a common surgical emergency marked by inflammation of the appendix, often due to blockage of the appendix lumen by fecoliths, lymphoid hyperplasia, or neoplasms. While various causes are known, appendicitis triggered by a foreign body (FB) is exceptionally rare. This case report highlights a rare presentation of appendicitis in a 32-year-old male with no significant medical history, who presented with acute lower right abdominal pain, fever, and vomiting. Initial evaluation suggested appendicitis, further supported by laboratory findings and diagnostic imaging revealing a retrocecal appendix with surrounding inflammation. Remarkably, an FB, a fish bone, was discovered lodged within the perforated appendix, elucidating the unusual etiology. Emergency laparotomy confirmed the diagnosis and facilitated prompt surgical intervention. This case underscores the importance of thorough evaluation and consideration of uncommon causes in patients presenting with acute abdominal pain, illustrating the critical role of detailed history-taking and clinical acumen in guiding management decisions and ensuring favorable patient outcomes.

## Introduction

Acute appendicitis ranks among the most frequent causes of surgical emergencies and admissions, with males facing a lifetime risk of 8.6% and females 6.7% [1]. Luminal obstruction triggers inflammation and infection, attributable to various factors such as fecal conglomeration (appendicolith), calculi, tumors, and lymphoid hyperplasia secondary to infection elsewhere and due to foreign body (FB) ingestion. Ingestion of foreign objects is more commonly observed in children but is a rare occurrence among adults, particularly among those with intellectual disability [1,2]. While most FBs pass through the gastrointestinal tract without causing complications, there is a risk of perforation with sharp objects or erosion and abrasion if the object remains lodged. Although uncommon, acute appendicitis can result from FBs, with an estimated prevalence of around 0.0005%, occurring in adults who inadvertently swallow foreign objects along with food the most common being fish bones and chicken bones [2]. When FBs enter the appendix, they trigger an inflammatory response, which may lead to perforation in some cases. 

Several scoring systems like the Alvarado score, Appendicitis Inflammatory Response Score (AIRS), and Adult Appendicitis Score aid in stratifying patients into low and high-risk categories, guiding management decisions [1]. Radiological tools such as ultrasound (US) and computed tomography (CT) play pivotal roles in diagnosis. Despite emerging evidence supporting antibiotic therapy for acute appendicitis, surgical removal remains the gold standard [1].

This case report highlights successful surgical treatment of a patient who ingested a fish bone along with food leading to inflammation and perforation of the appendix. The experience underscores the importance of prompt intervention and adds to the existing literature on this rare phenomenon.

## Case presentation

A 32-year-old male presented to the emergency department complaining of sudden lower right abdominal pain, accompanied by fever and vomiting. He denied any alterations in bowel habits or rectal bleeding. His medical and family histories were unremarkable. During examination, his pulse rate was elevated at 122 beats per minute, blood pressure measured 130/90 mmHg, temperature was 102°F, and respiratory rate stood at 22 breaths per minute. Abdominal examination revealed diffuse tenderness with guarding. Rectal examination did not reveal any bleeding or masses.

**Figure 1:**
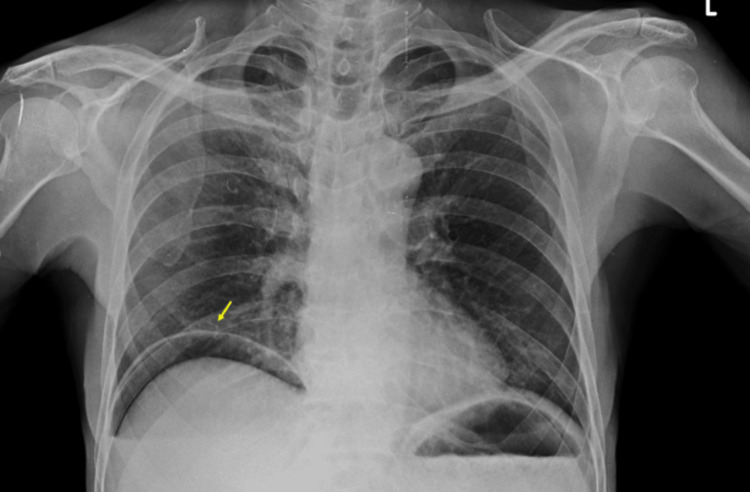
Chest radiograph frontal view showing air under the right diaphragm (yellow arrow), evident of pneumoperitoneum.

Initial assessment and diagnostic imaging suggested the possibility of appendicitis. Laboratory investigations indicated leukocytosis (white blood cell count of 18,000/mm³) and a hemoglobin level of 12.6 g/dL. Renal function tests and serum electrolytes were within normal parameters. However, the C-reactive protein (CRP) level was elevated up to 50 mg/DL, alongside elevated levels of procalcitonin (0.78 ng/ml). The elevated levels of CRP and procalcitonin suggested in favor of complicated appendicitis suggesting the possibility of perforation. A plain X-ray revealed the presence of air under the diaphragm, indicating pneumoperitoneum (Figure 1).

**Figure 2:**
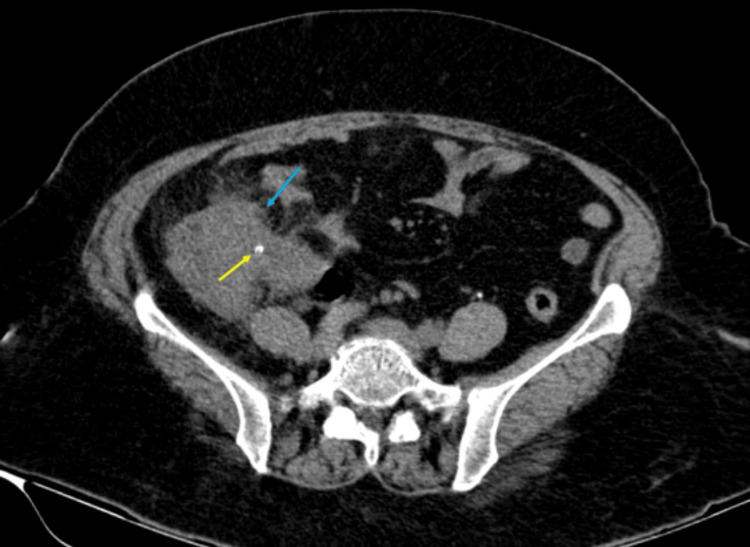
CT of the abdomen axial section showing appendicular mass with perforation (blue arrow) and tiny hyper-dense focus within the appendicular mass (yellow arrow). CT: Computed tomography

The patient was immediately taken up for ultrasonography of the abdomen which showed RIF probe tenderness and dilated tubular, non-compressible appendix with significant surrounding inflammatory changes and suggested further imaging since the patient was non-compliant. Further details were discerned initially through CT abdomen imaging, which revealed a dilated, thickened appendix situated pelvic in position, accompanied by surrounding fat stranding indicative of acute appendicitis. Additionally, a tiny hyperdense structure generally suggestive of an appendicolith was noted; however, in this case, suspicion of a fish bone (consistent with the patient's history) was raised. This was seen within the inflamed appendix, raising suspicion of perforation due to FB ingestion. Pneumoperitoneum was evident, suggesting perforation. No other acute intra-abdominal abnormalities were noted (Figure 2).

**Figure 3:**
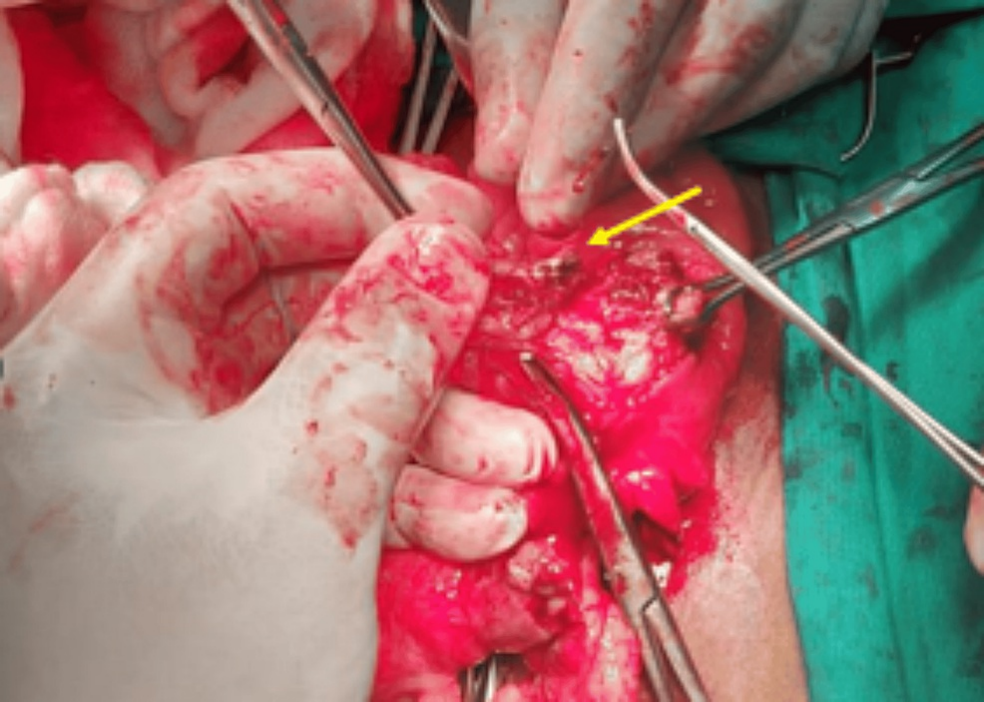
Intra-operative image demonstrating the fish bone (yellow arrow) within the inflamed appendix causing subsequent perforation.

**Figure 4:**
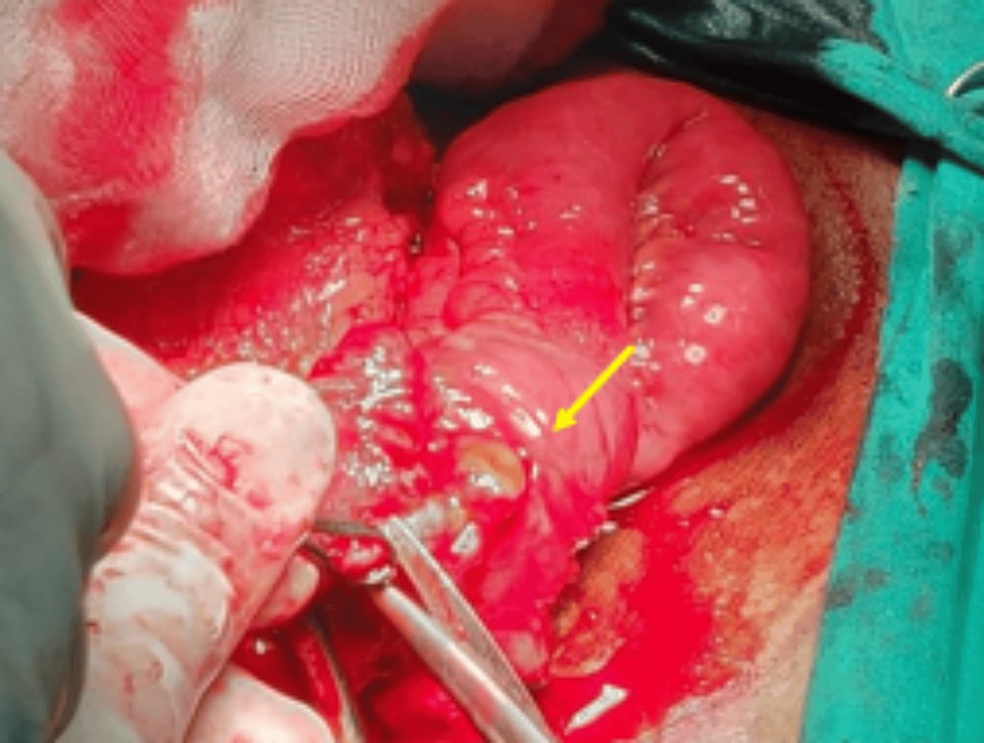
Intra-operative image showing the perforated appendicular lumen at the base of appendix (yellow arrow).

**Figure 5:**
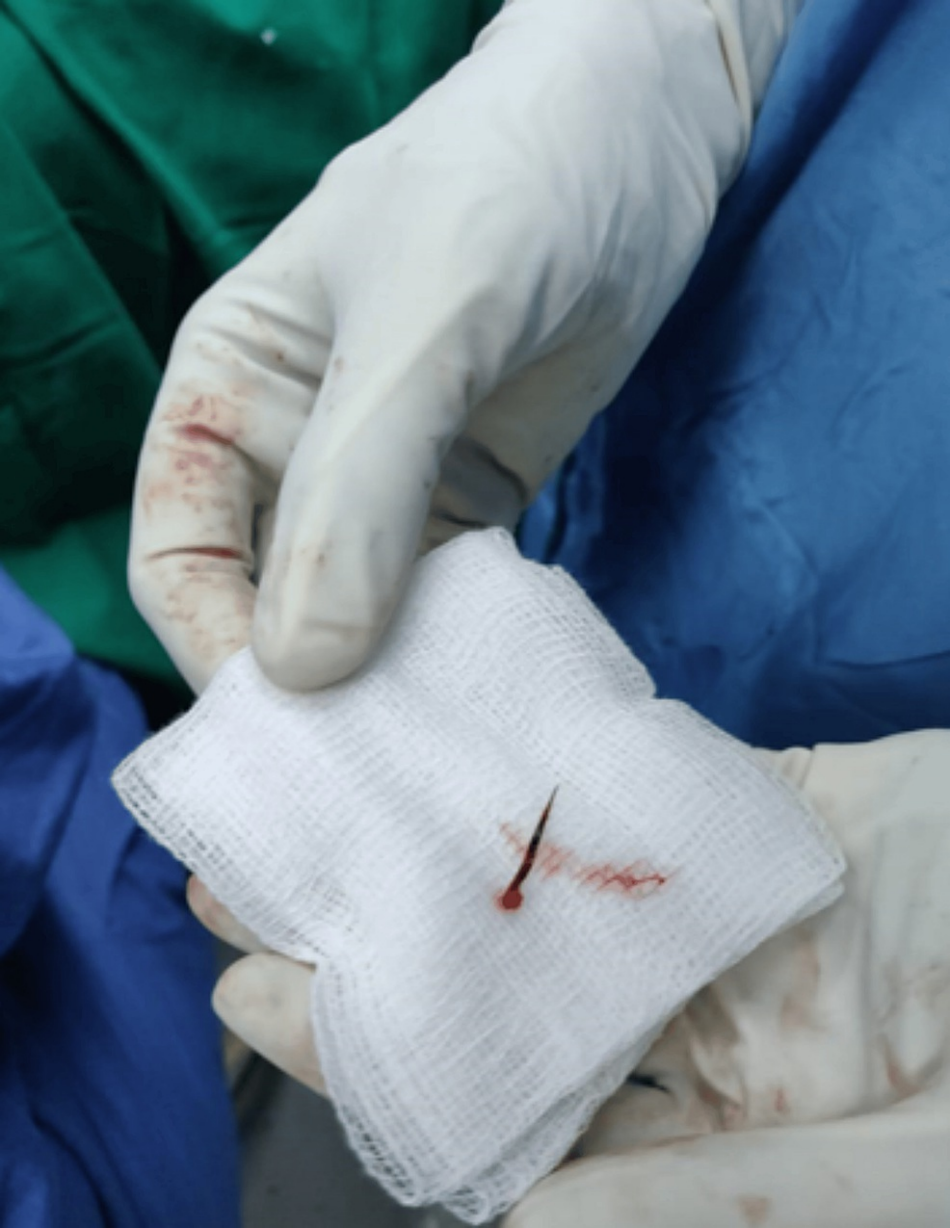
Post-operatively, the fish bone that caused the appendicular perforation is demonstrated.

With the presence of pneumoperitoneum, the patient underwent an emergency lower midline laparotomy. Intra-operatively, a mass was identified in the right iliac fossa region, with the appendix found in the pelvic position. The appendix was perforated at the base with a FB, a fish bone, lodged within the inflamed appendix (Figures 3, 4). The fish bone was removed and demonstrated separately (Figure 5). This confirmed the CT findings to be consistent with the FB rather than a simple appendicolith, thus elevating the importance of proper history taking and correlation.

Following surgery, the patient experienced an uncomplicated recovery and was discharged on the fourth post-operative day. Subsequent inquiry revealed the patient's accidental ingestion of a fish bone one week prior to the onset of symptoms.

This case underscores the importance of thorough evaluation in cases of acute abdominal pain, even when symptoms appear typical of appendicitis. Additionally, it highlights the significance of obtaining a detailed history, as it can provide crucial insights into the underlying cause of the condition.

## Discussion

FBs causing appendicitis are exceptionally rare, and identifying them often poses a diagnostic challenge. In this case, the patient's lack of awareness regarding ingesting a foreign object hindered early investigation. The mechanism by which the FB leads to appendicitis involves luminal obstruction and subsequent inflammation.

A perforated appendix due to an FB presents a unique and potentially life-threatening scenario. Etiologically, FBs, such as ingested objects or appendicoliths, can obstruct the appendiceal lumen, leading to inflammation, ischemia, and eventual perforation. Recent research has highlighted FBs as a potential factor in the onset of acute appendicitis [2]. Clinical assessment typically involves classic signs of appendicitis, including right lower quadrant pain, fever, and leukocytosis, but may also include features of peritonitis if perforation has occurred.

The ingestion of foreign objects typically does not lead to gastrointestinal issues like acute appendicitis, and they often pass through the digestive system within a week with a complication rate of less than 1% [3]. The timing of symptoms and the entry of an FB into the appendix are influenced by factors such as the object's size and shape, as well as the appendix's anatomical location [3]. Sharp and elongated FBs are more common and are prone to causing appendix perforation and subsequent peritonitis.

Due to the cecum's dependent position and relatively low motility, FBs tend to settle there. The likelihood of an FB entering the appendiceal lumen depends not only on the tightness or openness of its orifice but also on the appendix's anatomical position. In cases where the appendix is retrocecal, the chances of an FB entering its lumen are nearly non-existent. Blunt FBs may remain inactive for a period since peristaltic movements are often insufficient to push them back into the cecal lumen [3,4]. They can stay immobile within the appendix without causing inflammation or may incite an inflammatory response, with or without perforation. The time interval between ingesting the FB and the appearance of symptoms can vary from hours to years [4].

Radiological evaluation plays a pivotal role in diagnosis, with CT being the preferred modality. CT scans can identify appendiceal inflammation, fluid collection, and the presence of an FB. Newer modalities such as magnetic resonance imaging are emerging as adjuncts, offering non-invasive alternatives in select cases.

Intervention typically involves surgical exploration, appendectomy, and removal of the FB. Minimally invasive techniques, such as laparoscopy, are preferred when feasible, offering shorter recovery times and reduced morbidity. Post-operative care includes antibiotics, monitoring for complications, and patient education to mitigate future risks. Early recognition and intervention are crucial in optimizing outcomes for patients with perforated appendicitis due to FBs [4,5].

Clinicians should maintain a high index of suspicion for unusual etiologies in cases of atypical presentations of appendicitis which include generalized abdominal pain, painful urination, flatulence, and abdominal distention stimulating bowel obstruction. Detailed patient history, meticulous imaging studies, and intra-operative exploration are crucial for accurate diagnosis and appropriate management. It underscores the significance of thorough evaluation, including detailed history-taking and consideration of rare causes, to ensure accurate diagnosis and appropriate management. Further studies and case reports documenting unusual presentations of appendicitis can contribute to our understanding of this condition and aid in refining diagnostic and management strategies.

## Conclusions

In conclusion, this case emphasizes the necessity of comprehensive assessment in instances of acute abdominal pain, even when symptoms initially suggest appendicitis. By meticulously evaluating clinical presentation, conducting appropriate diagnostic imaging, and obtaining a thorough patient history, we can effectively identify uncommon causes such as FB ingestion leading to appendiceal perforation. Prompt recognition and surgical intervention are pivotal in achieving favorable patient outcomes, as demonstrated in this case. Furthermore, this report underscores the importance of vigilance and meticulousness in medical practice to ensure accurate diagnosis and timely management of potentially life-threatening conditions.
